# Implementation of an Individual + Policy, System, and Environmental (I + PSE) Technical Assistance Initiative to Increase Capacity of MCH Nutrition Strategic Planning

**DOI:** 10.1007/s10995-022-03435-0

**Published:** 2022-05-21

**Authors:** Dena R. Herman, Amy Blom, Angela Tagtow, Leslie Cunningham-Sabo

**Affiliations:** 1 Department of Community Health Sciences, UCLA Fielding School of Public Health, Los Angeles, CA 90095 United States; 2 Colorado School of Public Health, Aurora, CO 80524 United States; 3 Äkta Strategies, LLC, Elkhart, IA 50073 United States; 4 Department of Food Science and Human Nutrition, Colorado State University, Fort Collins, CO 80523-1571 United States

**Keywords:** I + PSE, MCH nutrition, Technical assistance, Capacity-building, Partnerships, Leadership

## Abstract

**Introduction:**

Childhood obesity disproportionately affects low-income women, children, racial/ethnic minorities, and rural populations. To effectively promote sustainable change, healthy eating and active living initiatives should apply individual plus policy, systems, and environmental (I + PSE) approaches.

**Methods:**

Four public health maternal and child nutrition teams selected through an application process participated in 12 months of technical assistance (TA) to develop action plans incorporating I + PSE in nutrition programming. TA included: (1) online modules; (2) community of practice (CoP) meetings; and (3) individual coaching sessions. Teams completed midpoint and endpoint surveys to assess TA knowledge and process outcomes. Semi-structured, in-depth interviews conducted post TA were transcribed and content analysis used to characterize themes and sub-themes.

**Results:**

Facilitators to implementing I + PSE approaches included TA delivery through online modules, participation in the CoP, and individual coaching to address barriers to implementation and leadership support. Barriers were time and funding limitations, working in isolation, and lack of infrastructure and self-efficacy. Co-learning helped TA teams overcome stagnancy and promote development of creative solutions. Teams recognized relationship-building as integral to systems development.

**Discussion:**

Lessons learned occurred across three main areas: relationships, capacity-building, and barriers encountered. Relationship formation takes time and is often not recognized as an asset impacting public health programing. Relationship direction – upstream, downstream, and lateral - affects ability to build organizational and systems capacity. While this study includes a small number of public health nutrition teams, this practice-based research highlights the value of I + PSE TA to tackle complex problems, with reciprocal, multisectoral support to enhance public health nutrition program impact.

**Supplementary information:**

The online version contains supplementary material available at 10.1007/s10995-022-03435-0.

## Significance:

***“What is already known on this subject?***

Childhood obesity is a complex and multifactorial public health challenge. Historically, there has been a focus on individual education to address childhood obesity, but literature demonstrates the complexities of the issue and need to address the issue through a social determinants of health lens.

***“What this study adds?***

While traditional MCH nutrition service delivery focuses on direct services, this practice-based research points to the value of I + PSE technical assistance initiatives to tackle complex public health problems such as childhood obesity with reciprocal, multisectoral support to leverage program impact for community and population benefit.

## Introduction

Childhood obesity is a complex and multifactorial public health challenge linked with a variety of health comorbidities expressed early in life that often persist into adulthood (Singh et al., 2008, Weiss and Caprio, 2005). To address complex public health challenges, there is a need for whole systems approaches that consider multifactorial causes and address the issue collaboratively across disciplines and sectors while engaging diverse stakeholders (Bagnall et al., 2019; Mabry and Bures, 2014). Historically, there has been a focus on individual education and behavior change to address childhood obesity, but literature demonstrates the complexities of the issue and need to address it through the social determinants of health (Yusuf et al., 2020). Social determinants of health are “the conditions in the environments where people are born, live, learn, work, play, worship, and age that affect a wide range of health functioning, and quality-of-life outcomes and risks (US Department of Health and Human Services, Office of Disease Prevention and Health Promotion, 2021).”

The US Department of Health and Human Services’ Maternal and Child Health Bureau (MCHB) works to improve MCH concerns like childhood obesity by providing ten essential services, and by providing competitive funding opportunities to training programs. These ten essential services are contextualized through the Public Health Services for MCH Populations: Title V MCH Services Block Grant Pyramid that provides a framework to comprehensively address needs of mothers and children in the United States: direct services, enabling services, and public health services and systems (US Department of Health and Human Services, 2020).

The policy, systems, and environmental (PSE) framework has risen in prominence in healthy eating and active living (HEAL) programming as a systems framework that robustly addresses the social determinants of health (Honeycutt et al., 2015). PSE approaches, when applied to obesity prevention, move beyond a focus on individual behavior and allow contextualization to communities and their specific needs (Boutain and McNees, 2013; Cheskin et al., 2017). Ample literature demonstrates that PSE approaches are more apt to be applied when there are technical assistance (TA) programs available. TA facilitates PSE approaches by supporting effective planning and programming, promoting critical thinking, facilitating teams’ learning, and helping organizations work more collaboratively (Hefelfinger et al., 2013). Le et al. (2016) describe characteristics of effective TA as programs that are content-driven, relationship-based, and adapted to the context of the organization along a continuum. The most successful programs prioritize feedback, awareness of context, flexibility, and engagement through tailoring of the program to the learner’s context (Chiappone et al., 2018).

**Fig. 1:**
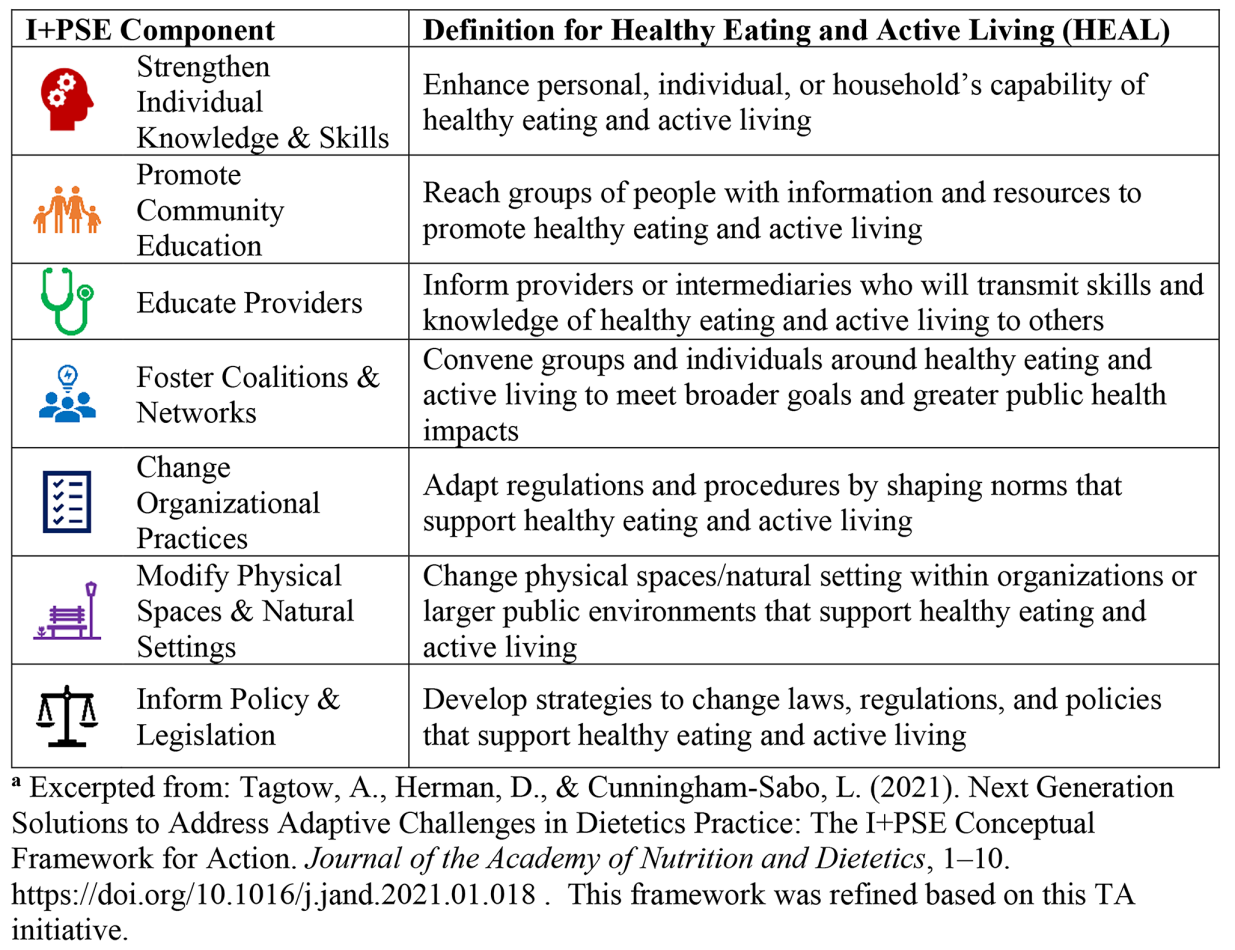
Seven Components of the I + PSE Model for Healthy Eating and Active Living^a^

A limitation of PSE approaches is they tend to de-emphasize the direct health care service delivery that is central to the Title V MCH Services Block Grant Pyramid. The novel Individual plus Policy, Systems, and Environment (I + PSE) Conceptual Framework for Action addresses this shortcoming by not only including the individual component but provides a multidimensional framework for addressing adaptive challenges (Heifetz, 2009) and driving sustainable and systemic impact (Tagtow et al., 2021). The Framework identifies root causes using a determinants of health lens that informs coordinated strategies across seven domains [Fig. 1]. Evaluation mechanisms must be equally dynamic and encompass the individual, practice, program, organizational, policy, and population levels. The Framework is adaptable to a range of complex nutrition and health issues including childhood obesity prevention (Tagtow et al., 2021).

This study evaluated the effectiveness of an I + PSE TA initiative with four public health nutrition teams in the Western United States. During this 12-month effort, teams developed I + PSE strategies and incorporated them into strategic action plans for nutrition programming. This analysis sought to understand the common facilitators, barriers, expectations, and outcomes of the TA effort and the integration of I + PSE approaches in policy, program planning, and operations.

## Methods

### Study sample

Teams were recruited for the TA initiative funded through a supplement to the UCLA Fielding School of Public Health MCHB Nutrition Leadership Training program. A request for proposal was sent to the 40 members of the Western MCH Nutrition Leadership Network (NLN), a network of State and territory nutrition leaders working in the thirteen states and territories west of the Rocky Mountains (https://mchnutritionpartners.ucla.edu/western-mch-nutrition-leadership-network/) and members of an Indian Health Service (IHS) dietitian’s listserve. Proposals were reviewed by a panel of experts in MCH, evaluation, American Indian/Alaskan Native populations, and PSE approaches. Proposals reflected action plans to address childhood obesity for one or more of each team’s rural and/or American Indian, or Alaska Native communities. Reviewers used an evaluation to score the proposals and select the teams. The four teams with the highest scored proposals were selected to participate in the TA initiative. Teams were comprised of mid- to senior-level public health professionals working in Title V^1^ and serving State or county government organizations or delivering clinical services. Teams included between one and seven members as selected by each of the applicants. All team leaders had master’s degrees in public health or nutrition science and were registered dietitian nutritionists.

## Technical assistance activities

Table 1 [Table 1] shows the I + PSE TA core components and timing of activities. Program components included: (1) Completion of the five-module Systems Approaches for Healthy Communities (SAHC) online training program through the University of Minnesota Cooperative Extension. The training modules guided teams to take action on the many PSE factors influencing individuals or families to make healthy choices; (2) An I + PSE workbook designed specifically for MCH practitioners to develop HEAL strategies using the seven components of the Framework (Fig. 1). Definitions for each component were tailored to a HEAL context and at the conclusion of the project, teams integrated the I + PSE strategies into new or existing plans; (3) Participation in monthly calls using a Community of Practice (CoP) approach to deepen knowledge and expertise through co-learning and resource sharing; and (4) Monthly individual coaching calls with the CoP facilitator. Using principals and information gained through learning components, teams developed an action plan for their individual settings. Final plans were reviewed together at the last TA meeting, where teams discussed their intentions for implementation.

**Table 1:** I + PSE Technical Assistance Core Components and Timing

Core Component	Activities	Timing/Time in Activity	Operational Details
Concept Acquisition: UMN SAHC online training modules	Registration for and completion of five modules on PSE approaches.	Month 2 – Month 6/ 5 hours	Upon completion of each module, reflection sheets and evaluation assessments are completed to measure comprehension and application of materials.
Concept Application: Workbook: Individual + Policy, System, and Environmental (I + PSE) Conceptual Framework for Action to Healthy Eating Active Living Initiatives (HEAL)^a^	Complete tailored action sheets in workbook: I + PSE Conceptual Framework for Action and HEAL strategies in local setting	Month 2 – Month 6/ 5 hours	Application of I + PSE approaches with team of MCH public health practitioners to local setting. Serve as building blocks for development of action plan.
Co-learning/Capacity Building: Participation in Community of Practice (CoP) Discussions	Conduct CoP group discussions and individual coaching	Month 2 – Month 12^b^/ 5–13 hours	Coaching and technical assistant sessions to discuss lessons learned and barriers encountered. These sessions took place once monthly for 6 months. Schedule is set by group. The CoP discussions support co-learning and resource sharing. Coaching sessions use systematic reflection and action learning as tools to support capacity building, iteration, and system change.
Midpoint/Endpoint Evaluation	Are these the surveys? Need to add anything here?	Months 3, 7/ 2 hours	ditto
Implementation: I + PSE Action Plan for Obesity Prevention (and other PH issues)	Development of action plan for application in local setting	Month 12/ 2–5 hours	Using principals and information gained through online modules, workbook with tailored action sheets, and community of practice discussions, teams develop, review and implement an action plan for local settings.

^a^ The link to the workbook developed for this TA initiative can be found on the Association of Maternal and Child Health Program’s (AMCHP) Innovation Hub in the practice handout at: https://www.amchpinnovation.org/database-entry/individual-policy-systems-and-environmental-approaches-technical-assistance/.

^b^ The CoP started in Month 1 with an introductory call and took place monthly from Month 2 – Month 6. At that time, calls continued bimonthly through Month 12. Individual coaching took place monthly and as requested throughout the 12-month TA initiative.

## Data Collection and Analysis

A mixed-methods approach was used for data collection and analysis. Data sources included midpoint and endpoint surveys, endpoint interviews, and draft and final action plans. Surveys were collected online through Qualtrics, had between nine (midpoint) and nineteen items (endpoint) and covered the following categories of content: dissemination of I + PSE educational materials, number and types of relationships formed, involvement in State action plans, and organizational readiness to advance I + PSE approaches (endpoint only).Survey analysis involved assessing learning progress of I + PSE concepts over the 12-month time frame and examining relationships formed using a collaboration framework (Taylor-Powell, 1998) to characterize the degree of collaboration based on an integration scale from 1 (low) to 5 (high) with relationship integration increasing as depth of relationship increases.

Semi-structured, in-depth interviews were conducted remotely over video conference at the endpoint with each of the four teams (see interview guide in Appendix 2). Items addressed expectations, outcomes, and factors that promoted or inhibited program success. Interviews were recorded, with prior verbal consent, and transcribed verbatim. Transcripts were coded line-by-line with thematic content analysis by two independent coders. Interview items were used to deductively derive the four primary themes (*a priori* codes): facilitators, barriers, expectations, and outcomes. A variety of subthemes were inductively derived (emerging codes) from the activity. Coders discussed each interview to reach consensus on subthemes. Text segments could be coded to more than one subtheme if appropriate (Tolley, 2016; Saldaña, 2021).

To understand the significance of each subtheme across interviews, a weighted score was developed by counting: (1) number of quotes represented within each subtheme for each interview; and (2) number of quotes represented overall for each subtheme across all four interviews. These two scores were summed for the weighted score, giving greater significance to subthemes that were represented across more interviews, and based on the premise that a subtheme represented once across the four interviews was more significant than a subtheme represented four times in the same interview. The scoring system was developed specifically for this manuscript but was adapted from methods from Creswell and Poth (2017), Creswell and Plano Clark (2017) and Saldaña (2021). Draft and final action plans were compared to the I + PSE framework for action and changes in the number of activities and shifts across categories were assessed.

Study methods adhered to COREQ, a checklist to ensure research quality, transparency, and reproducibility (Tong et al., 2007). This study was approved by the IRB (UCLA Office of the Human Research Protection Program, IRB #20-001084).

## Results

Team members were between 20 and 64 years of age with a third of the total 14 team members from the four teams between 30 and 39 years. Three team members were Latinx, three African American, five White, and three American Indian. Team members worked in a range of public health programmatic areas including nutrition, breastfeeding, physical activity, early childhood education, education leadership, nursing and SNAP-Ed. Each of the teams had individuals who served in a coordinator or supervisory role.

Team 1’s plan focused on improving HEAL opportunities in early childhood education by taking a systems-level approach to include updates to licensing standards and training for licensed childcare and family home childcare providers. Team 2 targeted informing the Title V needs assessment process with particular focus on childhood obesity in American Indian populations. Team 3’s plan was to create a Regional Coalition of community-based organizations, health departments, and schools to develop a comprehensive plan of action to address childhood obesity with an American Indian health care center as their base. Team 4 focused on addressing upstream perinatal causes of later childhood obesity by identifying best practices for healthy weight gain during pregnancy.

### Results: Survey Data

Surveys (Appendix 1a and 1b) were delivered online through Qualtrics at the midpoint of the TA initiative (Month 4) and the endpoint (Month 12) to understand teams’ dissemination and application of I + PSE concepts, individual and organizational readiness to change, and understanding the number and types of partnerships developed during the TA opportunity. To characterize collaborative relationships, relationship integration was evaluated on a scale of 1 (low) to 5 (high) (Taylor-Powell, 1998).

Teams increased their efforts to disseminate I + PSE information from survey midpoint to endpoint by 52.9%. They disseminated and applied I + PSE concepts by identifying opportunities to share the UMN modules and I + PSE workbook with colleagues upstream (e.g., supervisors and upper management) (Team 3) and downstream (e.g., local health jurisdictions) (Team 2), creating coalitions with new partners (Team 4), and providing trainings for childcare providers (Team 1). At midpoint, all teams except Team 3 had plans to contribute to their State/local strategic plans, but by the endpoint all teams identified activities to do so. Team 4 contributed to nutrition, physical activity, breastfeeding and PSE, while Team 2 conducted the State’s needs assessment for their public health staff.

Individual and organizational readiness to change was assessed at the endpoint survey. Teams 2 and 4 reported theirs as “high”, Team 1 as “very high,”, and Team 3 rated individual readiness as “high” and organizational readiness as “low.” They attributed this to their organization’s lack of awareness and participation in I + PSE training to date. All teams except Team 1 reported gaining confidence to talk about I + PSE with others in their workplace due to participation in the TA initiative. Team 1 reported already feeling confident because of organizational support to include I + PSE approaches.

Although the number of relationships decreased from midpoint to endpoint, the proportion of relationships shifted from a greater proportion at a lower integration to a greater proportion at a higher integration, indicating an increase in deeper relationships at endpoint. For example, from survey midpoint to survey endpoint, communication-level relationships decreased from 30.9 to 0%, while cooperation (10.2–30%) and collaboration (12.8–20%) increased [Table 2].

**Table 2:**
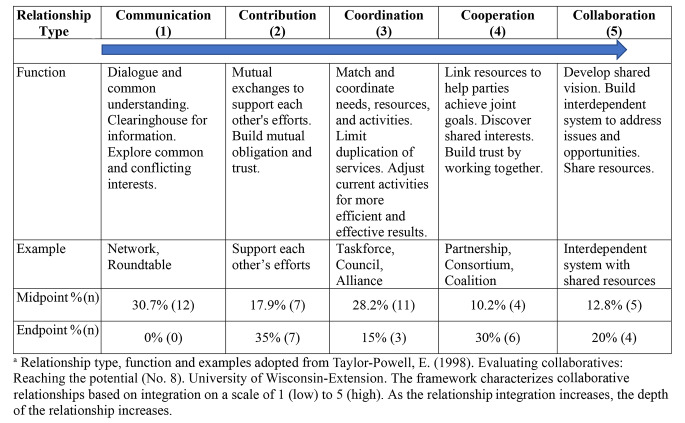
Assessment of Relationship Integration from Midpoint to Endpoint of the Technical Assistance Initiative^a^

### Results: qualitative interviews

The major themes of the qualitative analysis include the facilitators, barriers, expectations, and outcomes that TA teams experienced during this initiative [Table 3].

**Table 3:** Facilitators, Barriers, Expectations and Outcomes Identified as Major Themes and Subthemes during Qualitative Endpoint Team Interviews

Theme	Subtheme (Weighted Score)	Interview Quote
Facilitators	Mentorship (12)	“The one-on-one calls were incredibly helpful. Those are where we got the most direct recommendations for our work, because in those situations I was able to talk specifically about projects and get her guidance and suggestions on how to move some of that forward (Team 1).”
	Learning Activities Broadened Thoughts (6)	“Going through the activities and the modules and the worksheets helped me identify areas where I could expand the work and do more coverage…it helps you think of new ways to engage new partners (Team 1).”
	Support: Co-learning with other teams (4)	“It’s good to have people in the field to talk to…Any of these things are great to talk with other states what they are doing (Team 4).”
	Support: Curricular (4)	“I thought this would be a great foundation we could all kind of proceed from so that we’re all starting on the same page and then we would have the support of people who knew more than we did on this topic (Team 3).”
Barriers	Uncertainty/Self Doubt (12)	“Part of it was that I thought originally I wasn’t going to apply because I didn’t think our early learning work had enough structure. I was worried we were unsure how we were going to move forward with it (Team 1).”
	Working alone difficult (11)	“I mean I did feel like I was kind of floundering for a while and part of it was because I was solo (Team 2).”
	Slow Progress/Took Longer (8)	“It would be nice if we knew this opportunity was available for let’s say five years…cause this coalition type thing is going to take a while to really get going, it may take five years for us to get to where we really want to be with it (Team 3).”
	Struggle to come together (8)	“The people that do all of our trainings we haven’t renewed their contracts. So everything got put on hold, doing more webinars (Team 4).”
Expectations	Guidance/Direction (7)	“When we learned of this technical assistantship, I thought it was a great opportunity for us to gain insight into what would be the best foundation for us to start with, in trying to eventually get to where our goal is (Team 3).”
	Expectations: Exceeded (Coaching, TA Activities) (6)	“I think that this whole experience… surpassed what I thought that we would be getting from it, because you are an entire team over there working on this, you know you are knowledgeable on this topic and gave really good support, um, in areas that we did not feel confident in so much so that when we started… (Team 3).”
	Resources: Future (4)	“So we first want to get a foundation here at WIC then include others and kind of expand, expand, expand so that we all have that common foundation. And then we can better sell the idea of this coalition (Team 3).”
	Resources: Knowledge and tools for impact (4)	“I think for me it was to gain some strong, effective tools to provide the services that we are currently providing, within our program, for me the youth wellness program and to learn more about what is effective and what will make a great impact in our program planning (Team 3).”
	Value of Relationships (4)	“I was shown that a lot of things that didn’t really feel like progress like relationship building… (and even just mapping out what is happening in and around early learning work) in our state (Team 1).”
Outcomes	Expansion (Realities and Possibilities) (27)	“There was a lot of interest. I have contact info at the county level for programs that I normally don’t have... (Team 3).”
	Areas for Improvement (16)	“It might have been good to have done the trainings—the modules as a group—as a webinar and then we had discussions. I did it with people here but there was always different people coming. You know, people wanted to come because everyone didn’t have matching calendars here it was hard even getting anyone to be on the call (Team 4).”
	TA Effects: TA moved Thinking Forward (14)	“I do think this experience really did challenge me to expand my thinking about my approaches in early learning work, um, and so I could see myself using that framework again (Team 1).”
	TA Effects: Deliverables from TA Project (14)	“We have created the brand—we have successfully created the sort of logo brand to identify all of these different projects as one body of work. And we have that approved through Department of Health which is great (Team 1).”

### Facilitators

The primary facilitators included: mentorship, how learning activities broadened thoughts, and support received. While mentorship was the most often mentioned subtheme, support, in terms of both co-learning and curricular activities were also highly rated. TA teams shared a common language through the I + PSE concepts they learned both during the CoP calls and individual coaching that helped them and their co-workers to be on the “same page” and gain more support for working together in their workplace. TA curriculum and activities, the credibility and validity of the I + PSE Conceptual Framework for Action, easily understandable modules and learning activities, and individual coaching also facilitated action plan development.

Upstream support, receiving support from managers or supervisors, and downstream support, interest and participation in activities from local health jurisdictions or county-level workers, were major facilitators. One respondent stated, “I am very supported by my program manager…She did participate in a couple of calls with “the coach” and she appreciated the assistance that “the coach” was able to provide to the early learning work (Team 1).”

The flexibility of the TA was a facilitator in meeting teams where they were at and tailoring TA specifically to their needs. A member from Team 2 stated, “you did not pigeon-hole us into this narrow focus or direction, with the realization that every team is different and that we all were going at different speeds and had different partners in place.” Additional facilitators included helpful pressure, nudges from TA leaders, and having I + PSE work funded.

### Barriers

Barriers encountered were uncertainty and self-doubt, difficulties working alone, slow progression of activities, and the struggle to come together to do I + PSE activities as a group. Although I + PSE concepts were not new for some TA teams, understanding how to implement them was not always clear, which led to difficulties knowing how to move forward. Recognition that relationship-building is a time-consuming process and how to include it as part of usual job tasks was also challenging. Team members who had to work by themselves or had to wait on others to move forward also expressed frustration making progress.

Three of the four teams struggled with finding co-worker or leadership support and found it difficult to move forward with I + PSE activities [Table 3]. One team stated, “I encouraged participation of other teammates and colleagues, but they just didn’t have the bandwidth (Team 2).” A lack of upstream support also proved to be a significant barrier in gaining traction for some of the teams.

Lack of capacity, whether organizational, financial, or time-related, was also cited as a barrier. A respondent stated that “you know when you have a small team, it’s really easy for us to try to do everything…sometimes it feels as if we have to do every piece of the puzzle (Team 3).” Teams found it hard to coordinate: “there was always different people coming. You know, people wanted to come [but] because everyone didn’t have matching calendars here it was hard even getting anyone to be on the call (Team 4).”

### Expectations

Expectations of the TA included provision of capacity-building resources such as guidance and direction to implement I + PSE approaches, acquisition of specific tools and knowledge to increase impact, the value of coaching, and appreciating the value of relationships.

Expectations did not always line up with reality: “our expectations were a little different than what was the end result for all of us. Things took longer. Part of that expectation was that it’s not like you can go through a linear process and every state is different (Team 2).” Teams also looked forward to connecting with other teams to learn about how to engage in network and coalition building - “any of these things are great to talk with other states to know what they are doing (Team 3).”

### Outcomes

All respondents described outcomes around the ability to expand the visions for their work - whether in new relationships or in new directions programmatically. The TA helped move “thinking forward” with respect to increasing understanding and interest in the I + PSE modules and confidence in I + PSE approaches among co-workers. Deliverables from the TA included: project branding, a website housing I + PSE resources, updated learning modules and trainings including I + PSE concepts, a position paper and a policy brief, evaluation efforts, and increased team knowledge of I + PSE approaches.

Relationship building was a key outcome and included relationships within teams and fostering external connections. The value of co-learning with other teams emerged as a significant subtheme. “I think any time you hear what other states are doing, it gives you insights into what you can do in your own [work], either with the current work you are doing or future work; so that’s really helpful and I appreciated being able to share our experiences with other states (Team 1).”

Teams found they gained better communication skills and encouragement from other teams. They appreciated the skillsets within their own teams and either gained or refreshed their public health skillsets. Additionally, teams found the TA helped them work more effectively, review career goals, and overcome stagnancy.

### Results: Action Plans

Teams submitted draft action plans at the beginning of the project period and final action plans at the end. Action plan activities were characterized by the I + PSE Framework components [Fig. 1] and draft plans were compared to final plans to evaluate quality and changes. One aspect of plan quality was the extent to which activities were included for each of the seven I + PSE components and to identify any movement in activities up the continuum from working with individuals to working across systems. Team 4 did not submit a final plan and is therefore not included in this analysis. A summary of the action plan activities and plan excerpts are provided in Table 4 [Table 4]. Teams varied greatly in their implementation of I + PSE activities depending on their prior experience with I + PSE approaches and amount of support received from supervisors (i.e., upstream support). Teams 1 and 2 had some experience with PSE approaches while Team 3 had very little. Team 1 had strong upstream support for TA activities, Team 2 had medium support and Team 3’s organization lacked the knowledge and experience to be able to support them.

**Table 4:** Initial and Final Action Plans Categorized by I + PSE Component based on the I + PSE Conceptual Framework for Action^a^

Definition for Healthy Eating and Active Living (HEAL)^b^ from I + PSE Conceptual Framework for Action	Total Activities Initial Plans	Total Activities Final Plans	Sample of Activities from Final Action Plans
Enhance personal, individual, or household’s capability of healthy eating and active living	1	7	• Early childcare education focus on increasing individual knowledge and skills. • Deepen I + PSE knowledge and skill of Title V staff through Systems Approaches for Healthy Communities. • Increase minutes of physical activity with individuals and families in clinical practice.
Reach groups of people with information and resources to promote healthy eating and active living	6	3	• Farm to ECE curricula have a community involvement and education component. (e.g., CACFP to Farm to ECE) • Incorporate nutrition, food security, breastfeeding and physical activity into Title V needs assessment process. • Work with school food service staff to increase children’s intake of nutritious foods.
Inform providers or intermediaries who will transmit skills and knowledge of healthy eating and active living to others	4	6	• Train Early Achievers Coaches. • Promote and facilitate Title V local grantees participation in Systems Approaches for Healthy Communities. • Work with elementary school teachers to decrease children’s consumption of sugary beverages at school.
Convene groups and individuals around healthy eating and active living to meet broader goals and greater public health impacts	12	12	• Early Learning Workgroup. • Leverage existing partnerships to participate in Title V needs assessment process. • Conduct Organizational Readiness to Change Survey.
Adapt regulations and procedures by shaping norms that support healthy eating and active living	4	2	• Developing trainings for early learning administrators on Healthy Eating and/or Breastfeeding policies. • Local Title V action plans incorporate PSE approaches in addition to the individual approaches.
Change physical spaces/natural setting within organizations or larger public environments that support healthy eating and active living	0	2	• Disseminate information on new grants for early learning programs to expand infrastructure, including modifying kitchens and outdoor spaces. • Existing policies and practices put in place to support healthy meetings, wellness at work, expression of breast milk, active transport.
Develop strategies to change laws, regulations, and policies that support healthy eating and active living	1	2	• Assist with implementation of new statewide licensing standards for early learning programs, which increased requirements for nutrition and physical activity. • Developed two White Papers (i.e. policy brief) on Breastfeeding and Food Security highlighting I + PSE approaches, illustrating nutrition as one of foundations for addressing social determinants of health.

^a^ Excerpted from: Tagtow, A., Herman, D., & Cunningham-Sabo, L. (2021). Next Generation Solutions to Address Adaptive Challenges in Dietetics Practice: The I + PSE Conceptual Framework for Action. *Journal of the Academy of Nutrition and Dietetics*, 1–10. 10.1016/j.jand.2021.01.018. This framework was refined based on this TA initiative. I + PSE framework components are shown in Fig. 1.

^b^ HEAL – Healthy Eating Active Living.

Team 1 increased the number of Individual Knowledge and Skills activities from none in their draft plan to two in their final plans, decreased the number of Community Education activities from three to one, increased their activities to Modify Physical Spaces and Natural Settings from none to one, while the number of activities in the other components remained the same. Team 1 completed most of the initial tasks they set out in their draft plans and started to engage in new tasks in their final plans. For example, in terms of Informing Policy and Legislation, in their initial plan, they served as subject matter experts for trainings to local health jurisdictions related to nutrition, physical activity, breastfeeding, and screen time. In their final plans they assisted with implementation of new statewide licensing standards for early learning programs, which involved increased requirements for nutrition and physical activity representing broadening of their policy work from the draft plan to the final plan.

Team 2 increased the number of activities in Individual Knowledge and Skills, Modify Physical Spaces and Natural Settings, and Informing Policy and Legislation from none in their draft plan to one in their final plan. The number of activities in the Promoting Community Education and Changing Organizational Practices components remained the same, while there was a decrease in activities to Foster Coalitions and Networks from four to three. Across all components, activities moved from being “assessment-focused” in the draft plan to being incorporated into various systems in the final plan. For example, with respect to Educating Providers, the initial activity was to conduct a thorough needs assessment to identify how to approach childhood obesity in a culturally sensitive manner, while the final plan included “leading a community of practice for specific priorities and developing an elevator speech about public health and I + PSE approaches and nutrition.” While there were no policy-related activities proposed in the draft plan, the final plan proposed development of two policy briefs on breastfeeding and food security highlighting I + PSE approaches and illustrating nutrition as one of the foundations for addressing SDOH.

Finally, Team 3 showed increased activity from their initial draft in Individual Knowledge and Skills from one to four activities, Promoting Community Education and Educating Providers from no activities to one activity in each. The number of activities in Fostering Coalitions and Networks and Changing Organizational Practices stayed the same as they had some challenges getting these activities started. They were not able to develop activities in Modifying Physical Spaces and Natural Settings or Informing Policy and Legislation in either the draft or final plans. Most of the work of this team focused on Fostering Coalitions and Networks to address childhood obesity among Native Americans. The TA and coaching supported them to delineate specific tasks needed to engage and assemble a coalition of practitioners. This included obtaining support and buy-in of the Community Services Division leadership, the organization’s management team and board, and conducting an organizational readiness to change survey. As the team with the least experience in I + PSE approaches, they had the greatest learning curve toward implementation.

## Discussion

During this 12-month TA initiative, four public health nutrition teams developed childhood obesity prevention action plans incorporating I + PSE approaches. The teams engaged in several learning activities including completion of five, PSE online modules, monthly participation in a CoP and monthly individual coaching sessions. Surveys at the midpoint and endpoint of the TA initiative were administered to assess the outcomes of this process. This evaluation demonstrated that the TA initiative resulted in team learning evidenced by action plans, and that integrating I + PSE approaches into existing efforts can strengthen the capacity of the nutrition workforce and nutrition initiatives.

Results showed that the TA promoted critical thinking, which enabled teams to formulate creative and adaptive approaches to address childhood obesity and to overcome commonly cited “stagnancy” and “uncertainty.” The guidance and resources provided facilitated team learning through a content-driven curriculum allowing teams to adapt what they learned to their individual organization’s context. The TA initiative integrated facilitated discussion and reflection in the CoP and coaching calls to engage teams and generate continual feedback. This enabled teams to work more collaboratively and to maximize opportunities to form relationships, identify areas for expansion, and produce deliverables. Research shows that TA activities that include coaching and interpersonal communication support, combined with webinars, TA calls, and action institutes, are instrumental for program success (Honeycutt et al., 2015 and Hefelfinger et al., 2013).

The meta-theme of relationships underlaid teams’ success and served to build organizational capacity. Results from interviews and action plan analysis demonstrated changes to relationship quantity and relationship quality while building relationships both internal and external to their teams. Over the course of the TA, surveys revealed that the number of relationships decreased, but the integration, or depth of relationships increased. This coincides with the I + PSE Conceptual Framework for Action stages of change (Tagtow et al., 2021) and demonstrates that as teams moved along the continuum of building systems, their “Phase 2 relationships to formulate and implement solutions” deepened – meaning they moved from Strengthening Individual Knowledge and Skills to Activating Intermediaries and ultimately Facilitating Partnerships and Multisector Collaborations.

Teams who experienced more upstream support (i.e., from their supervisors and managers), experienced greater success implementing I + PSE changes; teams lacking that support, or working alone, found it more difficult to implement or gain traction for I + PSE change. Honeycutt et al. (2015) describes the importance of capacity and partnership building for PSE interventions prior to intervention planning and implementation. Successful communities were characterized as having broad, grass-roots community participation (Hefelfinger et al., 2013). Inclusion of these partners in conversation as well as sharing I + PSE resources takes time but are key elements to building organizational and downstream capacity (Bagnall et al., 2019).

While garnering upstream support was not always characterized as a marker of success by team members, it became evident that having and building strong relationships, whether upstream, laterally, or downstream was crucial. Upstream support is most effective when relationships between those proposing systems change engage with policy actors early in the process to develop relationships based on trust and shared understanding (Lloyd-Williams et al., 2020). For teams, this was a learned process and as TA sessions progressed, teams leveraged their current relationships and formed new ones to build coalitions and networks to make more policy-driven decisions. Teams also recognized the importance of lateral relationships, mostly within their departments, to gain support and momentum for the efforts. Bagnall et al. (2019) suggest that to make system-wide changes, engaging in specific activities to develop and maintain effective relationships both within and between organizations is important. Forming lateral relationships also provides the basis for reciprocal support for idea exchange and problem-solving to overcome barriers.

Fostering coalitions and networks remained a core focus of teams throughout the 12-month TA effort. While coalition-building is not often seen as a measure of “success” and is challenging to quantify, building and fostering these relationships are important in the work of I + PSE change approaches. Development of coalitions and networks has been described as one of the key factors in successfully facilitating organizational change (Kegler et al., 2015). Coalitions and networks can provide important and varied connections to people from different organizations. This helps prepare a foundation for consensus building and the impetus needed to make organizational changes. Establishing greater collaboration among organizations enables them to work together for the health of the whole system rather than focusing on technical fixes to individual parts (Senge et al., 2015 and Tagtow et al., 2021). As was demonstrated in this project, some teams moved their network and coalition efforts forward to not only make changes at the organizational level, but also move up the “spectrum of prevention” to modify physical spaces and settings as well as drive toward policy change.

The strengths of this study are in the diversity of the teams included, the content-driven, tailored TA, and the mixed methods systematic evaluation. The four teams had varied experiences with I + PSE approaches – some with little to no experience and others who had training during their graduate education. The teams came from four different states in the Western region, addressing different aspects of childhood obesity with ethnically, racially and geographically diverse populations. The teams themselves included a wide range of public health practitioners including nutritionists, early childcare experts, and nurses. The TA was tailored specifically to MCH-practitioners, addressed the local team context and learners’ individual needs through the CoP and coaching, and coaching sessions used systematic reflection and action learning as tools to support capacity-building and system change.

This study also has limitations. First, the sample size is small with only four teams, making it difficult to generalize the findings. However, there are efforts to share more practice-based evidence to make local experiences known and to build from these experiences. Brownson et al. (2018) state more research is needed that responds to practitioner’s needs and circumstances and that more “tacit knowledge” or “colloquial evidence” is needed to understand these needs. While evidence from four Western-state nutrition teams is not expected to change the direction of evidence-based practice, these data help to shape the narrative toward further capacity-building efforts, scope of funding streams, and additional research. Second, the TA was implemented for 12 months, thereby limiting the opportunity to follow teams to better understand how the I + PSE approaches perform longitudinally. As one team noted, “it would be nice to evaluate after five years to see what has been done as change takes time.” Team members are part of a Western-region leadership network, making it possible to track their progress, at least anecdotally, and continue to share lessons learned and build on those lessons with other network members. Finally, the midpoint and endpoint survey questions were not taken from validated instruments but were constructed from a general literature review and input received from the funding program officer.

In summary, singular, technical solutions have been shown to garner limited impacts for complex and adaptive challenges such as those encountered in addressing childhood obesity. Targeting activities at multiple levels helps to ensure that a support network is formed to move public health initiatives upstream together. While evidence from this I + PSE TA initiative is limited, there are very few examples in the scientific literature of initiatives of this type with the MCH nutrition workforce. The results from this TA initiative point in the direction of implementing multidimensional strategies supported by multisector partners using a social determinants of health lens to develop more effective and sustainable public health solutions. More data are needed to substantiate this path forward and to be able to draw more definitive conclusions for MCH public health practice.

## Electronic supplementary material

Below is the link to the electronic supplementary material.

Supplementary Material 1

## Data Availability

Data are available upon request.

## Footnotes

1 Title V is the only Federal program that focuses solely on improving the health of all mothers and children. Title V is a partnership with State MCH and Children with Special Health Care Needs programs, reaching across economic lines to support core public health functions such as resource development, capacity and systems building, population-based functions such as public information and education, knowledge development, outreach and program linkage, technical assistance to communities, and provider training (AMCHP, 2022).

## Code Availability

N/A.
