# Supplementary material for: Implementation of an Individual + Policy, System, and Environmental (I + PSE) Technical Assistance Initiative to Increase Capacity of MCH Nutrition Strategic Planning
Source: Matern Child Health J. 2022 May 21;26(Suppl 1):216–28. doi: 10.1007/s10995-022-03435-0 (PMC9482593; doi:10.1007/s10995-022-03435-0)
Supplement: Supplementary file 1 — Supplementary Material 1 [file 10995_2022_3435_MOESM1_ESM.docx]

**Appendix 1a. Midpoint Survey Questions**

| 1.) How many people have accessed your I+PSE educational (informational) materials on your website (if you have one)? |
| --- |
| 2.) How many people received your educational/informational materials (distribution of written materials) for example, through email messages or mailings? |
| 3.) How many people have you made aware about your project through face-to-face meetings or phone conversations? |
| 4.) Who have you reached out to about your project? Please list the position job title and organizations here: |
| 5.) Based on your responses to the “Value of Relationships” worksheet above (page 20, module 3 in workbook) and thinking about the situation now: |
| 5a.) How many partners do you have working with you on this project? |
| 5b.) On what types of activities are you partnering? |
| 5c.) There are five types of relationships based on the definitions used in the Figure 2 worksheet above (page 22, module 3 in workbook). How many of each of these types of relationships do you currently have? |
| 6.) List the position/job titles of the people and their organizations that you are partnering/collaborating with now: |
| 7.) Based on your work in this project, are you contributing to your State action plan? If yes, how? |
| 8.) Based on your work in this project, are you contributing to another State's action plan? Whose? What are you sharing with them? |
| 9.) Please share anything else you'd like us to know (e.g. successes, challenges, next steps). |

**Appendix 1b. Final Survey Questions**

| 1.) How many individuals have you made aware of your I+PSE project? list their job position title, organization, and position in relation to you (either downstream, upstream, or lateral). |
| --- |
| 2.) With how many individuals have you shared the UMN modules license? |
| 3.) Have you developed any new I+PSE educational or informational materials to share with others? |
| 4.) Please describe the material(s) and audience(s), and add website link below: |
| 5.) Below, list the internal partners/organizations and their position titles that you are working with on your I+PSE project(s). Identify the extent of the current relationship using the key below: Extent of relationships with 1 being least formal to 5 being most formal: 1 = Communication (network, roundtable); 2 = Contribution (support each other's efforts); 3 = Coordination (Taskforce/Council/Alliance); 4 = Cooperation (Partnership/Consortium/coalition); and 5 = Collaboration (interdependent system with shared resources) |
| 6.) Below, list the external partners/organizations and their position titles that you are working with on your I+PSE project(s). Identify the extent of the current relationship using the key below: Extent of relationships with 1 being least formal to 5 being most formal: 1 = Communication (network, roundtable); 2 = Contribution (support each other's efforts); 3 = Coordination (Taskforce/Council/Alliance); 4 = Cooperation (Partnership/Consortium/coalition); and 5 = Collaboration (interdependent system with shared resources) |
| 7.) Based on your I+PSE project activities, are you contributing to or do you plan to contribute to your State/Organization's action plan? |
| 8.) Describe how you are or plan to contribute to your State/Organization's action plan: |
| 9.) What steps need to be taken to integrate the I+PSE framework and/or strategies into the action plan? |
| 10.) Have you shared your I+PSE activities with any other states? |
| 11.) Provide the name of the state, the name of the organization, and what you have shared: |
| 12.) On a scale of 1 - 5 (with 1 = very low readiness and 5 = very high readiness), what is your organization's current level of readiness to advance I+PSEs within your initiatives: |
| 13.) On a scale of 1 - 5 (with 1 = very low readiness and 5 = very high readiness), what is your level of readiness to advance I+PSEs within your initiatives: |
| 14.) Over the course of the project, do you feel you have met your expectations of individual and organizational readiness to change? |
| 15.) Explain the reasons why you have or have not met your expectations of individual and organizational readiness to change: |
| 16.) Following this experience, has your team gained confidence in talking to others about the benefits of I+PSE approaches? |
| 17.) Explain the reasons your team has or has not gained confidence in talking to others about the benefits of I+PSE approaches: |
| 18.) What are your next steps with your I+PSE initiatives? |
| 19.) Please share with us anything else about your team's experiences with the MCH Nutrition I+PSE technical assistance project: |

**Appendix 2. Post TA Semi-Structured Interview Questions**

Think back to when you first heard about this I+PSE TA opportunity and as you prepared your proposal.

1. What were your expectations then? How do those compare with your actual experience?
2. What have you gained from participating in this TA activity?
3. What was the most useful?
4. What has your team gained?
5. What was missing?
6. What could we have done differently?
7. Will this change your efforts in the future? If so, how?
8. What are your next steps?
9. How can we improve the technical assistance and engagement approach for the future?

**Appendix 3. COREQ Checklist (screenshots)**

**
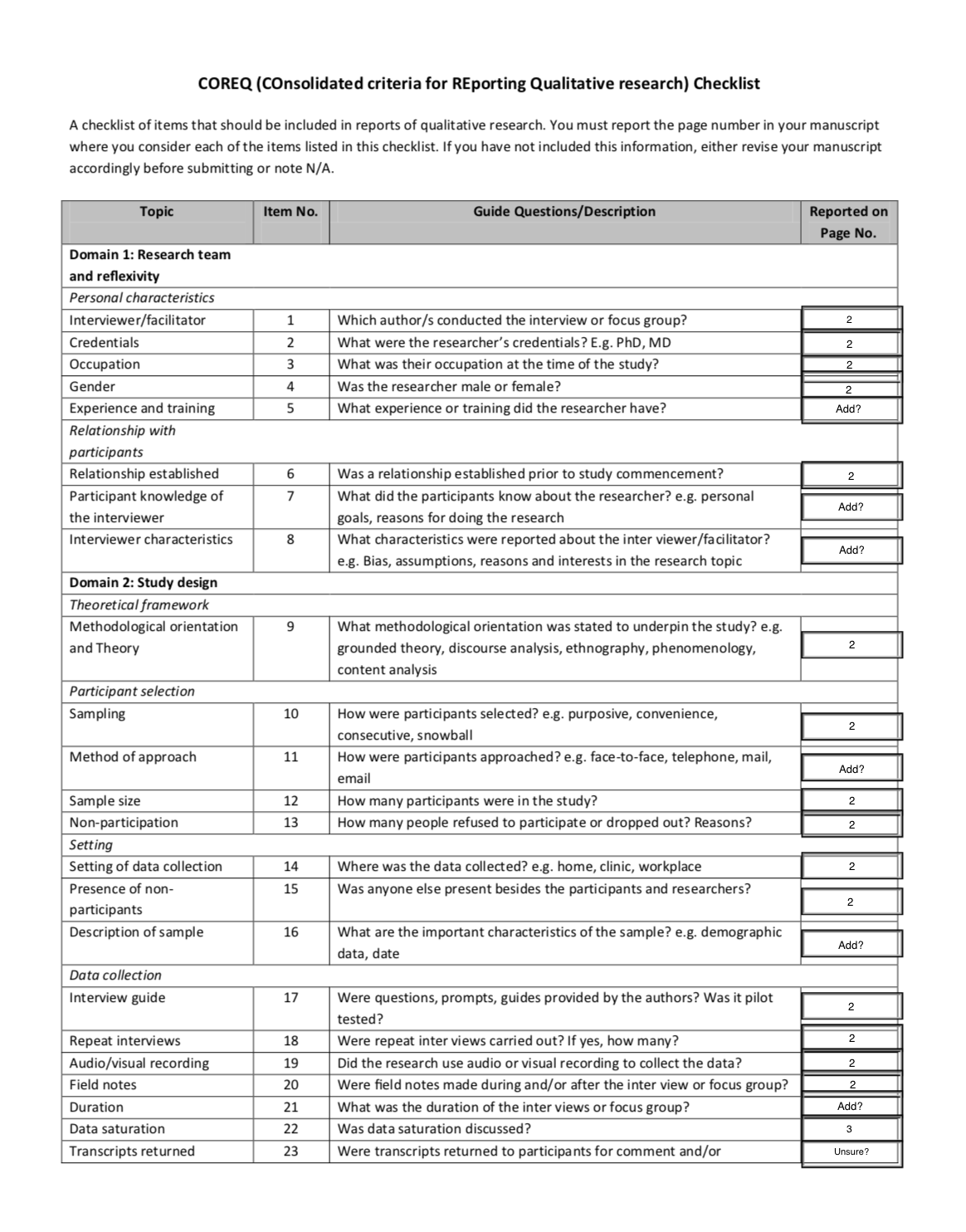
**

**
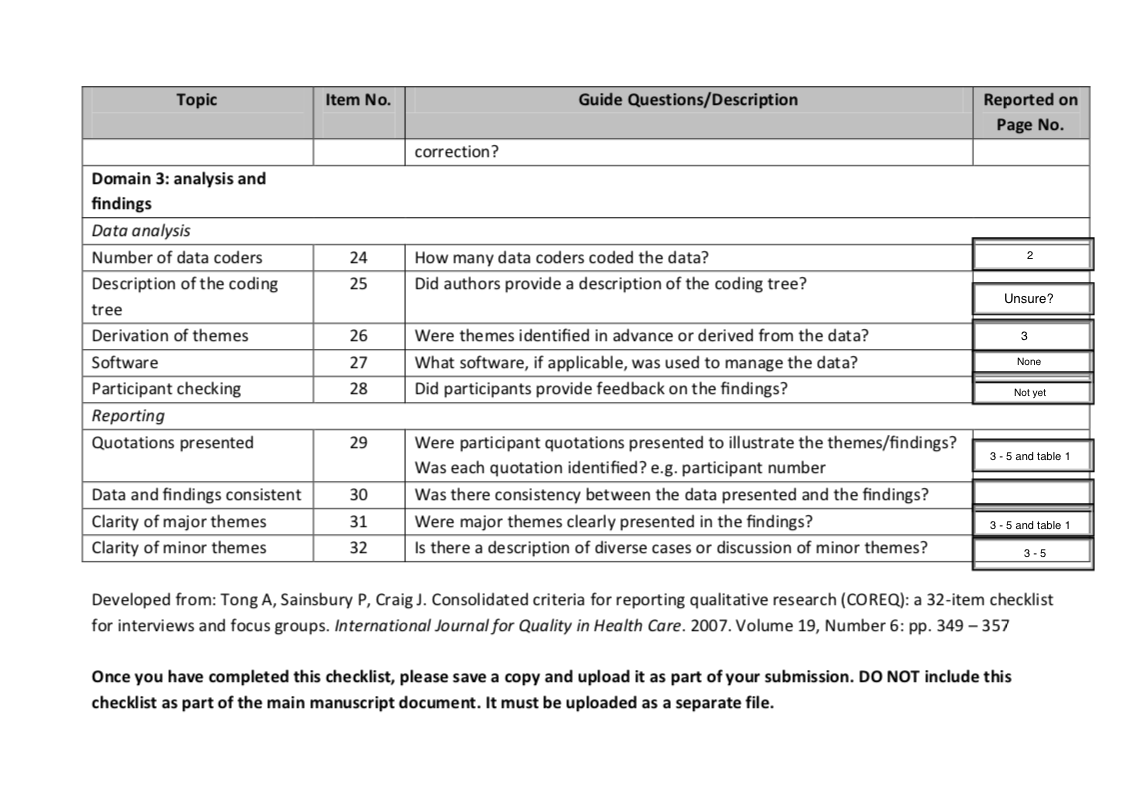
**
